# Influenza vaccination of pregnant women in Paris, France: Knowledge, attitudes and practices among midwives

**DOI:** 10.1371/journal.pone.0215251

**Published:** 2019-04-25

**Authors:** Paul Loubet, Catherine Nguyen, Espérie Burnet, Odile Launay

**Affiliations:** 1 Inserm, F-CRIN, Innovative clinical research network in vaccinology (I-REIVAC), Paris, France; 2 Inserm, CIC 1417, Paris, France; 3 Université Paris Descartes, Sorbonne Paris Cité, Paris, France; 4 AP-HP, Département de maladies infectieuses, CIC Cochin Pasteur, Hôpital Cochin Broca Hôtel-Dieu, Paris, France; 5 Ecole de sages-femmes Pitié Saint Antoine, Université Pierre et Marie Curie, Sorbonne Université, Paris, France; 6 Centre Hospitalier Intercommunal de Créteil, Maternité, Créteil, France; University of Campania, ITALY

## Abstract

**Introduction:**

In France, midwives have been authorized to prescribe vaccines since 2016. Yet vaccination coverage among pregnant women remains low. Understanding the knowledge, attitudes and practices of midwives regarding influenza vaccination could help improve coverage.

**Methods:**

A cross-sectional survey was conducted in 2017 among midwives practicing in the public and private sectors in Paris using an online questionnaire. Multivariate logistic regression analysis of the data was conducted.

**Results:**

The response rate was 31% (n = 208/669). Overall, knowledge of influenza vaccine recommendations and of vaccine safety and effectiveness was high except regarding new-born immunity and influenza vaccine characteristics. Only 10% of midwives systematically prescribed the vaccine. Reported influenza vaccine uptake among midwives was 39%.

**Conclusion:**

Efforts to improve the knowledge of midwives regarding the safety and effectiveness of vaccinating pregnant women in order to prevent influenza infection in newborns are necessary. Increasing vaccine uptake in both midwives and pregnant women will require adjusting education strategies.

## Introduction

Pregnant women and infants younger than 6 months are at high risk of developing severe influenza, which can cause cardiopulmonary complications and death [1,2]. Immunization against influenza during pregnancy has proven its effectiveness not only in mothers but in newborns as well, thanks to the passive transplacental transfer of antibodies [3–7]. However, although the WHO and French national guidelines recommend seasonal influenza vaccination in pregnant women at any trimester [8], coverage remains low [9,10]. Indeed, only 7,4% (95% Confidence Interval (CI): 6,9–7,9) of pregnant women were vaccinated in 2016, and only 24,9% (24,2–25,7) of them reported having been offered the vaccine by a health care provider [11].

Reasons for non-vaccination among pregnant women are known to include safety concerns for themselves and their child, lack of awareness and limited risk perception [10,12]. This underlines the need for improving women’s awareness of the risks of influenza infection and their understanding of immunity [13,14].

Midwives play a key role in the prevention of infectious diseases by informing and educating their patients. In France, they have been authorized to prescribe and administer vaccines to pregnant women, newborns and their relatives since 2016. Yet, although they have become new players in the effort to increase immunization coverage, their perceptions of vaccination and their practice in the field have only been little explored in France or in Europe. Investigating these factors could help improve influenza vaccine coverage among pregnant women. With this objective in mind, this study aims to explore the influenza vaccination-related knowledge, attitudes and daily practice of the midwife population in Paris, France.

## Methods

### Study design and population

A cross-sectional survey was conducted from October 1st to December 31st 2017 among midwives practicing in Paris, France (n = 669), using a standardized questionnaire administered online and distributed *via* email. Participants worked in public or private hospital maternity wards (n = 462), in child and maternal protection centers (n = 29) and in free-lance practice (n = 178). All were in active practice and registered with the Paris College of Midwifes.

### Questionnaire design

Two experts in the field of vaccine hesitancy (one infectious diseases specialist and one social science epidemiologist) were interviewed in order to assess the content validity of the questionnaire. They were asked to comment on the order, response scaling and grammatical structure of each item; to review the questionnaire overall; and to assess each item based on four criteria (relevancy, clarity, simplicity, and necessity). Questions were added or removed based on their evaluation and recommendations. Pilot study was conducted to test the questionnaire among fifteen students from the Pierre et Marie Curie University school of midwifery.

### Data collection (See S1 File)

The questionnaire was sent to practicing midwives by email, *via* supervisors in each of the maternity wards and child and maternal protection centers, and *via* the Paris College of Midwifes to reach those working free-lance. A reminder was sent 5 weeks later and questionnaire collection ended on 31 December, 2017. The questionnaire consisted of 25 multiple-choice questions, divided into three sections. Section one included items on socio-demographics (age, gender), practice characteristics (setting, graduation year), knowledge of influenza risks (for the mother and the fetus), of vaccination during pregnancy (recommendations, vaccine type and composition, vaccine safety and efficacy) and of vaccination in general (self-evaluated as limited, average or high). Section two addressed their attitudes towards influenza vaccination (patient education, vaccine prescription and/or administration, reasons for not implementing recommendations, exposure to information campaigns). Finally, in section three, participants were asked whether they had been vaccinated during the 2016/2017 influenza season and to describe the reasons why they had or had not received the vaccine. Completion of the questionnaire was estimated to take about ten minutes.

### Ethics

Participation in the survey was voluntary. A short paragraph was included at the beginning of the questionnaire to inform participants of the study’s objectives and of the confidentiality of their responses. Consent was considered obtained by virtue of questionnaire completion. Data was collected anonymously and participants had the right to access their answers. The Paris College of Midwives and the National Data Protection Authority (*Commission Nationale de l’Informatique et des Libertés*), which is responsible for ethical issues and for the protection of individual data, approved the survey and the methods used.

### Statistical analysis

Raosoft software was used to calculate sample size based on the following requirements: 5% margin of error, 95% confidence level, 50% response distribution and a total population of 669 (the total population of practicing midwives in Paris). A sample size of 245 was found to be statistically appropriate.

Continuous variables were considered as means and standard deviations (SD) or medians and interquartile ranges (IQR), and categorical/binary variables as numbers and percentages (n, %). The normality of the variables was assessed using both graphic (histograms) and numerical (Shapiro-Wilk test) methods.

Vaccine proposition was considered as a binary variable (never/sometimes vs. often/always). The associations between vaccine proposition (outcome) and exposure variables were evaluated in univariate analysis using logistic regression. The Wilcoxon rank sum test and Fisher’s exact test were used, as appropriate, to test for statistical significance (p<0.05).

A logistic regression model was built using a backward stepwise approach to include covariates with a p value <0.2 in univariate analysis. The same methods were used to assess variables associated with vaccine prescription.

Missing data were excluded from the analysis. All analyses were performed using Stata (V14, Copyright 1996–2014 StataCorpLPt).

## Results

### Population characteristics (Table 1)

Overall, 208 midwives responded to the questionnaire (response rate: 31% (208/669). Median age was 34 (IQR 28–43.5); 99% (n = 206) were female, and their median professional experience was 10 years (IQR: 4.5–19). In terms of practice setting, 66% (n = 137) worked in hospital maternity wards, 18% (n = 38) worked free-lance and 5% (10/208) in child and maternal protection centres. The remaining 11% worked in two different settings.

**Table 1:** Characteristics of the midwives who participated in the survey.

	Total N = 208
***Background***	
Women, n (%)	206 (99%)
Median age, years (IQR)	34 (28–43.5)
Practice setting, n (%)	
Public or private hospital maternity wards	137 (66%)
Free-lance practice	38 (18%)
Child and maternal protection centres	10 (5%)
Public or private hospital maternity wards and free-lance practice	21 (10%)
Public or private hospital maternity wards and Child and maternal protection centres	2 (1%)
Median years of experience, n (IQR)	10 (4.5–19)

### Knowledge

Most participants knew of the risks associated with influenza infection during pregnancy both for the mother (191/208, 92%) and for the foetus (188/208, 91%). They were also aware that vaccination against influenza is recommended during pregnancy (190/208, 91%) and can be administered at any trimester (155/208, 82%).

They agreed with the fact that the vaccine is effective (160/208, 76%) and safe (152/208, 73%) in pregnant women. Yet, fewer than half (97/208, 47%) knew that the vaccine will also protect the new-born through passive immunity thanks to the transplacental transfer of immunoglobulins.

Their knowledge of influenza vaccine characteristics was more limited. Although 71% (148/208) knew the vaccine is inactivated, only 52% (108/208) were aware that it does not contain an adjuvant. Overall, 63% of respondents considered their general knowledge of vaccination to be average (132/208), 32% thought it was limited (66/208) and 5% high (10/208). Self-reported knowledge of influenza vaccination was similar, with 53% rating it as average (111/208), 37% as limited (77/208), and 9% as high (20/208). More experienced midwives reported a greater degree of knowledge (p<0.01).

### Attitudes

Only 22 midwives (11%) systematically prescribed the influenza vaccine to their patients, 40% of whom administrated the vaccine themselves. Overall, 22% (46/208) of respondents never discussed influenza or the associated pregnancy-related risks with their patients during the flu season and 19% (40/208) never informed their patients that a vaccine was available. Among those who did not systematically suggest influenza vaccination (76% (158/208)), the main reasons given were forgetfulness (n = 80) and lack of time (n = 48). About one third of those working in hospital were aware that their institution had an influenza vaccine protocol (64/188, 34%) and 52% reported having seen a hospital-wide influenza vaccination campaign (108/208).

There was no difference in vaccine recommendation or administration rates between practice settings. In multivariate analysis, the presence of a vaccine protocol in the practice setting, high self-evaluated knowledge of influenza vaccination and being vaccinated against influenza were found to be associated with a higher likelihood of prescribing the vaccine **(see Table 2)**.

**Table 2:** Factors associated with vaccine proposition.

	Vaccine proposition to patients (often/always) N (%)	OR (95%CI)	p-value	aOR (95%CI)	p-value
***Total***	83/207 (40%)				
Sex			0.517		
Women	83/205 (40%)	-	-		
Men	0/2 (0%)	-	-		
Age			**0.013**		
[20–30[	26/74 (35%)	1	-		
[30–40[	20/67 (30%)	0.8 [0.4–1.6]	-		
[40–50[	21/39 (54%)	2.1 [1.0–4.7]	-		
[50–60]	16/27 (59%)	2.7 [1.1–6.6]	-		
Practice setting, n (%)			0.1		
Public or private hospital maternity ward	49/136 (36%)	1	-		
Free-lance practice	15/38 (39%)	1.1 [0.5–2.4]	-		
Child and maternal protection centres	4/10 (40%)	1.2 [0.3–4.4]	-		
Public or private hospital maternity wards and free-lance practice	14/21 (66%)	3.5 [1.3–9.4]	-		
Public or private hospital maternity wards and Child and maternal protection centres	1/2 (50%)	1.8 [0.1–4.4]	-		
Professional experience			**0.003**		
<10 years	29/99 (29%)	1			
≥ 10 years	54/108 (50%)	2.4 [1.4–4.3]			
General knowledge of vaccination			**0.001**		
Limited	16/65 (25%)	1			
Average	60/131 (46%)	2.5 [1.3–5.0]			
High	7/10 (70%)	7.1 [1.7–3.0]			
Knowledge of influenza vaccination			**<0.001**		**<0.001**
Limited	10/76 (13%)	1		1	
Average	55/111 (49%)	6.5 [3.0–14.0]		4.0 [1.7–8.9]	
High	18/20 (90%)	59.4 [12.0–295.7]		45.2 [8.7–235.1]	
Exposure to influenza vaccination campaign			**0.006**		
No	30/99 (30%)	1			
Yes	53/108 (49%)	2.2 [1.2–3.9]			
Presence of an influenza vaccination protocol			**<0.001**		**<0.001**
No	36/124 (29%)	1		1	
Yes	39/64 (61%)	3.8 [2.0–7.1]		3.5 [1.6–7.7]	
Influenza vaccine uptake			**<0.001**		**0.002**
No	29/126 (23%)	1		1	
Yes	54/81 (67%)	6.6 [3.5–12.4]		5.3 [2.4–11.7]	

### Self-reported Vaccine uptake (Table 3)

Influenza vaccine uptake among the midwives surveyed in this study was 39% (95%CI 33–46). The most frequent reasons for non-vaccination were “not being worried about catching influenza” (33%), “fear of side effects” (28%), “concerns about vaccine effectiveness” (13%) and “not having been offered a vaccine” (13%). There was no difference in vaccine uptake by practice setting. In multivariate analysis, only moderate/high self-evaluated knowledge of influenza vaccination was found associated with greater vaccine uptake. Midwives who had received the flu vaccine themselves were more likely to suggest their patients get vaccinated.

**Table 3:** Distribution of vaccine uptake among participants.

	Vaccine uptake N (%)	OR (95%CI)	p-value	aOR (95%CI)	p-value
***Total***	82/208 (39%)				
Sex			0.8		
Women	81/206 (39%)	1	-		
Men	1/2 (50%)	1.5 [0.1–25.0]	-		
Age			0.1		0.355
[20–30[	23/74 (31%)	1	-	1	
[30–40[	27/67 (40%)	1.5 [0.7–3.0]	-	1.9 [0.6–6.8]	
[40–50[	22/40 (55%)	2.7 [1.2–6.0]	-	1.9 [0.4–9.0]	
[50–60]	10/27 (37%)	1.3 [0.5–3.3]	-	0.8 [0.2–4.6]	
Practice setting, n (%)			0.179		0.864
Public or private hospital maternity ward	54/137 (39%)	1	-	1	
Free-lance practice	11/38 (29%)	0.6 [0.3–1.4]	-	0.4 [0.2–1.1]	
Child and maternal protection centres	4/10 (40%)	1.1 [0.3–3.8]	-	0.7 [0.2–2.2]	
Public or private hospital maternity wards and free-lance practice	11/21 (52%)	1.7 [0.7–4.3]	-	0.6 [0.2–2.8]	
Public or private hospital maternity wards and Child and maternal protection centres	2/2 (100%)	1	-	1	
Professional experience			**0.024**		**0.485**
<10 years	31/99 (31%)	1		1	
≥ 10 years	51/109 (51%)	1.9 [1.1–3.4]		1.3 [0.7–2.3]	
General knowledge on vaccination			**0.001**		**0.948**
Limited	15/66 (23%)	1			
Average	63/131 (48%)	3.2 [1.7–6.3]			
High	4/10 (40%)	1.5 [0.3–6.4]			
Knowledge on influenza vaccination			**<0.001**		**<0.001**
Limited	12/77 (16%)	1		1	
Average	59/111 (53%)	6.1 [3.0–12.6]		5.8 [2.8–12.1]	
High	11/20 (55%)	6.6 [2.3–19.4]		6.0 [2.0–18.1]	
Exposure to an influenza vaccination campaign			0.686		
No	38/100 (38%)	1			
Yes	44/108 (41%)	1.1 [0.6–1.9]			
Presence of an influenza vaccination protocol			0.321		
No	45/124 (36%)	1			
Yes	28/64 (44%)	1.4 [0.7–2.5]			
Recommendation of influenza vaccination to pregnant patients			**<0.001**		**0.185**
Never	7/63 (11%)	1		1	
Sometimes	20/61 (33%)	3.9 [1.5–10.1]		2.7 [0.9–8.6]	
Often	32/48 (67%)	16 [6.0–43.0]		8.6 [1.5–48.9]	
Always	22/35 (63%)	13.5 [4.8–38.4]		5.0 [0.7–33.9]	
Prescription of influenza vaccine to pregnant patients			**<0.001**		**0.127**
Never	11/73 (15%)	1		1	
Sometimes	21/59 (36%)	3.1 [1.4–7.2]		1.2 [0.4–3.6]	
Often	33/53 (62%)	9.3 [4.0–21.7]		1.5 [0.3–7.7]	
Always	16/22 (73%)	15.0 [4.8–46.8]		4.4 [0.5–36.8]	

OR: Odds Ratio; aOR: adjusted Odds Ratio

## Discussion

The results of the cross-sectional survey conducted among midwives practicing in Paris, France, showed that most midwives had a good understanding of influenza and related risks during pregnancy and of vaccine safety and effectiveness in pregnant women. Knowledge was more limited regarding the composition of the influenza vaccine itself and its ability to protect infants. Results also showed low rates of influenza vaccine proposition and prescription to pregnant women and low vaccine coverage among midwives.

The self-evaluated level of knowledge regarding the risks of influenza both for the foetus and for the mother and of vaccine’s safety and effectiveness and its recommendation during pregnancy was found to be high. Vishram *et al*. found similar levels of understanding in 2,939 English midwives in 2015 [15]. Yet, interestingly, only 47% of respondents knew that influenza vaccine during pregnancy protects the new-born, also similar to Vishram *et al*.’s findings. This is an important point as this argument can be used when suggesting vaccination to pregnant women. More than half of the participants in the survey thought that the seasonal influenza vaccine contained an adjuvant. This is also to be underlined, as adjuvants are often wrongly considered to be associated with adverse events.

More importantly, only 17% systematically recommended their patients be vaccinated and 10% systematically prescribed the vaccine. A higher level of knowledge, the existence of a vaccination protocol and being vaccinated against influenza were associated with higher offer and prescription rates. These findings are similar to those of Massot *et al*. who surveyed 917 student and professional midwives in France in 2017, and reported that only 23.5% declared having administered an influenza vaccine to pregnant women and that 51.5% were in favour of vaccinating pregnant women [16]. The attitudes of midwives towards influenza vaccine in France seem to be different from those in England, where 73% indicated that they routinely recommended the vaccine to pregnant women [15].

Despite being aware of national and international recommendations, Parisian midwives appeared reluctant to offer and prescribe the influenza vaccine, particularly those with shorter professional experience. This may be due to a lack of adequate training and information, as stated by nearly 60% of the midwives surveyed in the Massot study, which also found that 88.3% considered themselves to be in a good position to take on the responsibility of prescribing vaccines.

It is well known that considerable gaps exist regarding the level of knowledge related to vaccination in pregnant women and that the most effective factor in increasing vaccine uptake is improving patient understanding of disease prevention, of vaccine safety and efficacy, and of existing recommendations. Studies conducted in Italy have underlined that this information should preferably be provided by health-care workers and should be tailored to education level, number of children and religious and cultural factors [17–20].

The suggestion of vaccination by health care providers has been shown to be strongly associated with higher vaccine uptake in pregnant women in France [10] and in Italy [17,18] and many studies have shown that the advice of healthcare providers is generally trusted [10,21]. It is important to emphasize that they could and should become key players in overcoming public mistrust regarding vaccines, as has been shown in several studies exploring vaccine hesitancy in children [22–24], young adults [25] and parents [26].

Furthermore, influenza vaccine coverage of the midwives in our sample was only 39%. This rate was similar to that found by Massot (37%) [16] but lower to that found by Vishram *et al*. in England (58%) [15]. Most of the unvaccinated midwives either stated that they did not feel at risk of catching influenza or were concerned about vaccine safety and/or effectiveness. The implications of low vaccination coverage among midwives are that those that are not vaccinated can spread influenza to their patients, and that vaccinated midwifes are more likely to recommend vaccination. As in the general population [21,27] and in other health care professionals [28,29], vaccine uptake and recommendation increased with greater knowledge of influenza vaccination and vaccination in general, which underlines the need for 1) improving initial and continuing education, 2) improving the implementation of vaccination protocols and 3) increasing the number and reach of information campaigns.

The main limitation of this study was that our sample may not have been representative. Indeed, only one third (31%) of the practicing midwives in Paris responded to the online questionnaire. This may have led to an overestimation of vaccination coverage due to the health-consciousness of participants and introduced a degree of self-selection bias since those who chose to participate may have differed in terms of lifestyle, health choices and knowledge from those who declined. Information on non-respondents was not sufficient to quantify non-response bias.

## Conclusion

The inclusion of midwives among health-care professionals authorized to prescribe vaccines was thought to lead to an increase in the influenza vaccination rate in pregnant women in France. Yet this study shows that, although the midwives surveyed were aware of vaccination recommendations and of their ability to prescribe, they did not seem to have made the leap of systematically recommending and prescribing the vaccine to their patients. Midwife training and education should emphasize the effectiveness of the influenza vaccine in infants and the safety of the vaccine’s components. Systematic vaccination protocols and information campaigns explaining the benefits of vaccinating health-care workers who care for pregnant women should be generalized.

## Supporting information

S1 File Questionnaire used in the study. English and French versions.
(DOCX)

## References

[1] Neuzil KM, Reed GW, Mitchel EF, Simonsen L, Griffin MR. Impact of Influenza on Acute Cardiopulmonary Hospitalizations in Pregnant Women. Am. J. Epidemiol. 1998;148:1094–102. 9850132 10.1093/oxfordjournals.aje.a009587

[2] Neuzil KM, Mellen BG, Wright PF, Mitchel EF, Griffin MR. The Effect of Influenza on Hospitalizations, Outpatient Visits, and Courses of Antibiotics in Children. N. Engl. J. Med. 2000;342:225–31. 10.1056/NEJM200001273420401 10648763

[3] Zaman K, Roy E, Arifeen SE, Rahman M, Raqib R, Wilson E, et al Effectiveness of Maternal Influenza Immunization in Mothers and Infants. N. Engl. J. Med. 2008;359:1555–64. 10.1056/NEJMoa0708630 18799552

[4] Madhi SA, Cutland CL, Kuwanda L, Weinberg A, Hugo A, Jones S, et al Influenza Vaccination of Pregnant Women and Protection of Their Infants. N. Engl. J. Med. 2014;371:918–31. 10.1056/NEJMoa1401480 25184864

[5] Tapia MD, Sow SO, Tamboura B, Tégueté I, Pasetti MF, Kodio M, et al Maternal immunisation with trivalent inactivated influenza vaccine for prevention of influenza in infants in Mali: a prospective, active-controlled, observer-blind, randomised phase 4 trial. Lancet Infect. Dis. 2016;16:1026–35. 10.1016/S1473-3099(16)30054-8 27261067 PMC4985566

[6] Steinhoff MC, Katz J, Englund JA, Khatry SK, Shrestha L, Kuypers J, et al Year-round influenza immunisation during pregnancy in Nepal: a phase 4, randomised, placebo-controlled trial. Lancet Infect. Dis. 2017;17:981–9. 10.1016/S1473-3099(17)30252-9 28522338 PMC5573632

[7] Thompson MG, Kwong JC, Regan AK, Katz MA, Drews SJ, Azziz-Baumgartner E, et al Influenza Vaccine Effectiveness in Preventing Influenza-associated Hospitalizations During Pregnancy: A Multi-country Retrospective Test Negative Design Study, 2010–2016. Clin. Infect. Dis. [Internet] [cité 2018 nov 20];Available from: https://academic-oup-com.gate2.inist.fr/cid/advance-article/doi/10.1093/cid/ciy737/5126390 10.1093/cid/ciy737 30307490

[8] Loubet P, Anselem O, Launay O. Immunization during pregnancy. Expert Rev. Vaccines [Internet] 2018 [cité 2018 mai 9];Available from: https://www.tandfonline.com/doi/full/10.1080/14760584.2018.1471988 10.1080/14760584.2018.1471988 29715051

[9] Ding H. Influenza Vaccination Coverage Among Pregnant Women—United States, 2016–17 Influenza Season. MMWR Morb. Mortal. Wkly. Rep. [Internet] 2017 [cité 2018 avr 5];66. Available from: https://www.facebook.com/cdcmmwr 10.15585/mmwr.mm6638a2 PMC5657675 28957044

[10] Loubet P, Guerrisi C, Turbelin C, Blondel B, Launay O, Bardou M, et al Influenza during pregnancy: Incidence, vaccination coverage and attitudes toward vaccination in the French web-based cohort G-GrippeNet. Vaccine 2016;34:2390–6. 10.1016/j.vaccine.2016.03.034 27013430

[11] Blondel B, Coulm B, Bonnet C, Goffinet F, Le Ray C. Trends in perinatal health in metropolitan France from 1995 to 2016: Results from the French National Perinatal Surveys. J. Gynecol. Obstet. Hum. Reprod. 2017;46:701–13. 10.1016/j.jogoh.2017.09.002 29031048

[12] Freund R, Le Ray C, Charlier C, Avenell C, Truster V, Tréluyer J-M, et al Determinants of Non-Vaccination against Pandemic 2009 H1N1 Influenza in Pregnant Women: A Prospective Cohort Study. PLoS ONE 2011;6:e20900 10.1371/journal.pone.0020900 21695074 PMC3114856

[13] Tong A, Biringer A, Ofner-Agostini M, Upshur R, McGeer A. A Cross-Sectional Study of Maternity Care Providers’ and Women’s Knowledge, Attitudes, and Behaviours Towards Influenza Vaccination During Pregnancy. J. Obstet. Gynaecol. Can. 2008;30:404–10. 18505664 10.1016/s1701-2163(16)32825-0

[14] Ditsungnoen D, Greenbaum A, Praphasiri P, Dawood FS, Thompson MG, Yoocharoen P, et al Knowledge, attitudes and beliefs related to seasonal influenza vaccine among pregnant women in Thailand. Vaccine 2016;34:2141–6. 10.1016/j.vaccine.2016.01.056 26854910 PMC4811693

[15] Vishram B, Letley L, Hoek AJV, Silverton L, Donovan H, Adams C, et al Vaccination in pregnancy: Attitudes of nurses, midwives and health visitors in England. Hum. Vaccines Immunother. 2018;14:179–88. 10.1080/21645515.2017.1382789 PMC5791587 29048989

[16] Massot E, Epaulard O. Midwives’ perceptions of vaccines and their role as vaccinators: The emergence of a new immunization corps. Vaccine 2018;36:5204–9. 10.1016/j.vaccine.2018.06.050 29970300

[17] D’Alessandro A, Napolitano F, D’Ambrosio A, Angelillo IF. Vaccination knowledge and acceptability among pregnant women in Italy. Hum. Vaccines Immunother. 2018;14:1573–9. 10.1080/21645515.2018.1483809 PMC6067873 29863958

[18] Napolitano F, Napolitano P, Angelillo IF. Seasonal influenza vaccination in pregnant women: knowledge, attitudes, and behaviors in Italy. BMC Infect. Dis. 2017;17:48 10.1186/s12879-016-2138-2 28068918 PMC5223411

[19] Jalloh MF, Bennett SD, Alam D, Kouta P, Lourenço D, Alamgir M, et al Rapid behavioral assessment of barriers and opportunities to improve vaccination coverage among displaced Rohingyas in Bangladesh, January 2018. Vaccine 2019;37:833–8. 10.1016/j.vaccine.2018.12.042 30642728 PMC10472973

[20] Massimi A, Rosso A, Marzuillo C, Prencipe GP, Soccio PD, Adamo G, et al Childhood vaccinations. Validation of a tool for measuring knowledge, attitudes and vaccine hesitancy in pregnant women. Epidemiol. Biostat. Public Health [Internet] 2017 [cité 2019 mars 15];Available from: http://ebph.it/article/view/12625

[21] Loubet P, Kernéis S, Groh M, Loulergue P, Blanche P, Verger P, et al Attitude, knowledge and factors associated with influenza and pneumococcal vaccine uptake in a large cohort of patients with secondary immune deficiency. Vaccine 2015;33:3703–8. 10.1016/j.vaccine.2015.06.012 26073016

[22] Smith LE, Amlôt R, Weinman J, Yiend J, Rubin GJ. A systematic review of factors affecting vaccine uptake in young children. Vaccine 2017;35:6059–69. 10.1016/j.vaccine.2017.09.046 28974409

[23] Napolitano F, Navaro M, Vezzosi L, Santagati G, Angelillo IF. Primary care pediatricians’ attitudes and practice towards HPV vaccination: A nationwide survey in Italy. PLOS ONE 2018;13:e0194920 10.1371/journal.pone.0194920 29596515 PMC5875794

[24] Bianco A, Mascaro V, Zucco R, Pavia M. Parent perspectives on childhood vaccination: How to deal with vaccine hesitancy and refusal? Vaccine 2019;37:984–90. 10.1016/j.vaccine.2018.12.062 30655175

[25] Napolitano F, Napolitano P, Liguori G, Angelillo IF. Human papillomavirus infection and vaccination: Knowledge and attitudes among young males in Italy. Hum. Vaccines Immunother. 2016;12:1504–10. 10.1080/21645515.2016.1156271 PMC4964675 27070042

[26] Napolitano F, D’Alessandro A, Angelillo IF. Investigating Italian parents’ vaccine hesitancy: A cross-sectional survey. Hum. Vaccines Immunother. 2018;14:1558–65. 10.1080/21645515.2018.1463943 PMC6067864 29641945

[27] Sevin AM, Romeo C, Gagne B, Brown NV, Rodis JL. Factors influencing adults’ immunization practices: a pilot survey study of a diverse, urban community in central Ohio. BMC Public Health [Internet] 2016 [cité 2018 nov 15];16. Available from: http://bmcpublichealth.biomedcentral.com/articles/10.1186/s12889-016-3107-9 10.1186/s12889-016-3107-9 PMC4877755 27216805

[28] Loulergue P, Moulin F, Vidal-Trecan G, Absi Z, Demontpion C, Menager C, et al Knowledge, attitudes and vaccination coverage of healthcare workers regarding occupational vaccinations. Vaccine 2009;27:4240–3. 10.1016/j.vaccine.2009.03.039 19481314

[29] Hulo S, Nuvoli A, Sobaszek A, Salembier-trichard A. Knowledge and attitudes towards influenza vaccination of health care workers in emergency services. Vaccine 2017;35:205–7. 10.1016/j.vaccine.2016.11.086 27919630

