# Supplementary material for: Influenza vaccination of pregnant women in Paris, France: Knowledge, attitudes and practices among midwives
Source: PLoS One. 2019 Apr 25;14(4):e0215251. doi: 10.1371/journal.pone.0215251 (PMC6483190; doi:10.1371/journal.pone.0215251)

**Influenza vaccination of pregnant women in Paris, France: knowledge, attitudes and practices among midwives**

**S1 File. Questionnaire used in the study. English and French versions**

1. ***Knowledge of influenza and influenza vaccine***
   1. *In your opinion, can an influenza infection during pregnancy cause complications for the mother?*
      1. Yes
      2. No
      3. Don’t know
   2. *In your opinion, can an influenza infection during pregnancy cause obstetric/fetal complications?*
      1. Yes
      2. No
      3. Don’t know
   3. *In your opinion, is vaccination against influenza recommended for pregnant women?*
      1. Yes
      2. No
      3. Don’t know
   4. *In your opinion, is vaccination against influenza effective in pregnant women?*
      1. Yes
      2. No
      3. Don’t know
   5. *In your opinion, is vaccinating pregnant women protective against influenza for newborns in their first months of life?*
      1. Yes
      2. No
      3. Don’t know
   6. *In your opinion, is vaccination of pregnant women against influenza safe?*
      1. Yes
      2. No
      3. Don’t know
   7. *What type of influenza vaccine can be used in pregnant women?*
      1. Live attenuated vaccine
      2. Inactivated vaccine
      3. Don’t know
   8. *In your opinion, does the seasonal influenza vaccine used in pregnant women contain an adjuvant?*
      1. Yes
      2. No
      3. Don’t know
   9. *When should the influenza vaccine be administered during pregnancy?*
      1. First trimester
      2. Second trimester
      3. Third trimester
      4. Any trimester
      5. Don’t know
2. ***Attitudes towards influenza vaccination and self-reported practice***
3. *Do you provide information on influenza to pregnant women during the flu season?*
   1. Always
   2. Often
   3. Sometimes
   4. Never
4. *Do you provide information to pregnant women on influenza vaccine recommendations during the flu season?*
   1. Always
   2. Often
   3. Sometimes
   4. Never
5. *Do you offer to vaccinate your patients against influenza?*
   1. Always
   2. Often
   3. Sometimes
   4. Never
6. *If not, what are the main reasons?*
   1. Lack of time
   2. I do not think it is my role
   3. Forgetfulness
   4. I did not know that influenza vaccination was recommended in pregnant women
   5. Other reason
7. *Do you prescribe the influenza vaccine to your patients?*
   1. Always
   2. Often
   3. Sometimes
   4. Never
8. *Do you administer the influenza vaccine to your patients?*
   1. Yes
   2. No
   3. Do not know
9. *Does your institution have influenza vaccination protocol?*
   1. Yes
   2. No
   3. Don’t know
10. *If so, do you think it may have had an influence on your practice?*
    1. Strongly agree
    2. Agree
    3. Disagree
    4. Strongly disagree
    5. Don’t know
11. *Has your institution ever run a promotion campaign to encourage influenza vaccination?*
    1. Yes
    2. No
    3. Don’t know
12. ***General knowledge of vaccination***
13. *How would you rate your general knowledge of vaccination?*
    1. High
    2. Average
    3. Limited
14. *How would you rate your knowledge of influenza vaccination?*
    1. High
    2. Average
    3. Limited
15. ***Influenza vaccine uptake and participant sociodemographics***
16. *Were you vaccinated against influenza during the 2016/2017 season?*
    1. Yes
    2. No
    3. Don’t know
17. *If so, what were the main reasons?*
    1. To protect myself
    2. To protect the people around me
    3. the vaccine was offered to me
18. *If not, what were the main reasons?*
    1. I have doubts about vaccine effectiveness
    2. I am afraid of the vaccine’s side effect
    3. Influenza doesn’t concern me
    4. I am against vaccination in general
    5. The vaccine was not offered to me
    6. Other
19. *In what setting do you work?*
    1. Public or private hospital maternity ward
    2. Free-lance practice
    3. Child and maternal protection centres
    4. Public or private hospital maternity wards and free-lance practice
    5. Public or private hospital maternity wards and Child and maternal protection centres
20. *What is the name of you Hospital maternity ward?*
    1. I work in free-lance or in Child and maternal protection centres
    2. Armand Trousseau
    3. Bichat-Claude Bernard
    4. Diaconesses
    5. Institut Mutualiste Montsouris
    6. Pitié-Salpétrière
    7. Lariboisière
    8. Les Bluets
    9. Robert Debré
    10. Necker
    11. Port Royal
    12. Saint Joseph
    13. Tenon
21. *What year did you graduate?*
22. *What is your age?*
23. *You are :*
    1. A man
    2. A woman


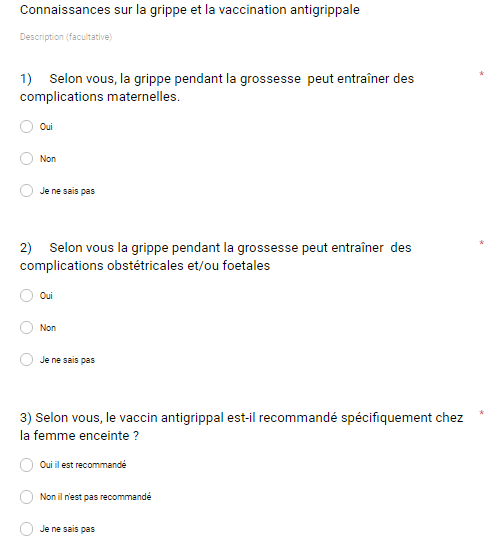


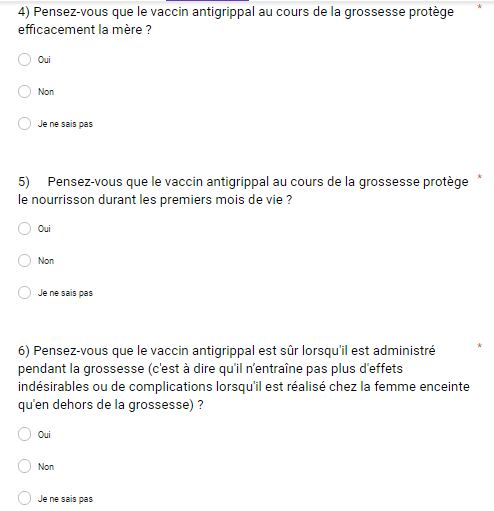

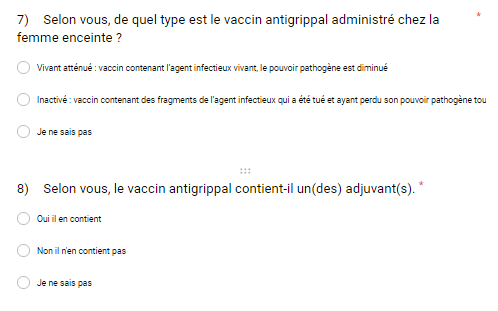


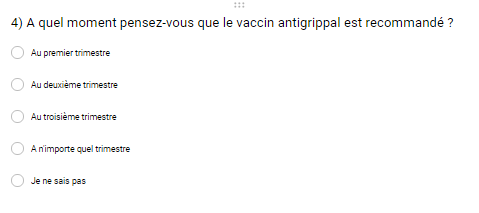


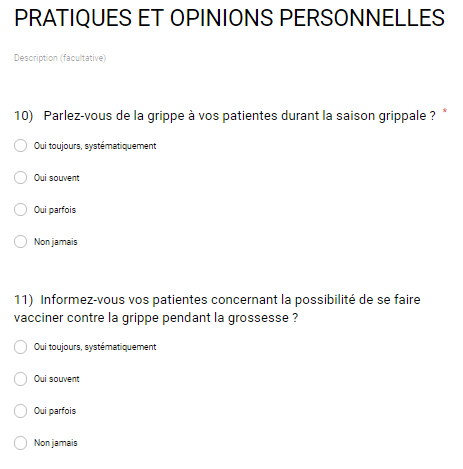


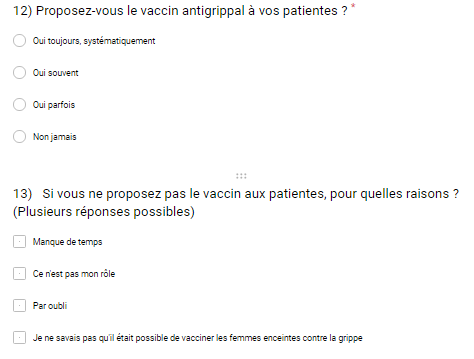


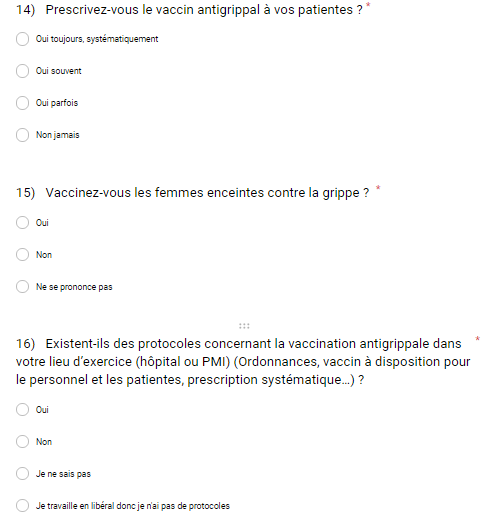


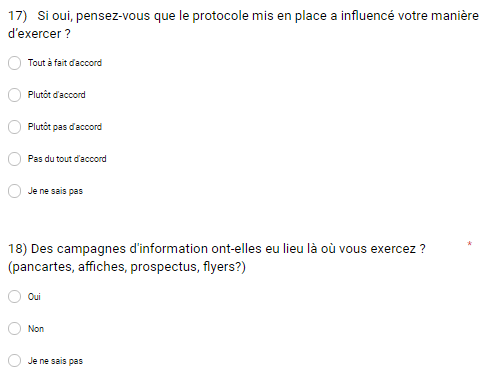


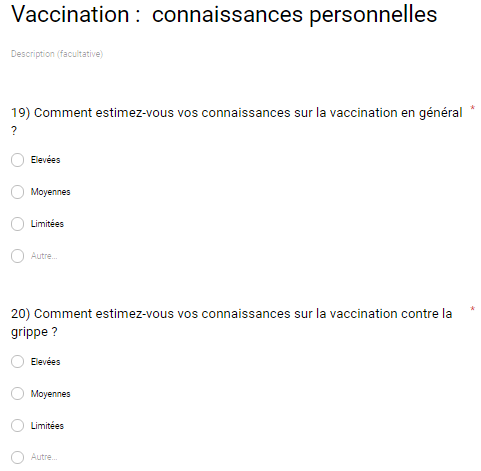


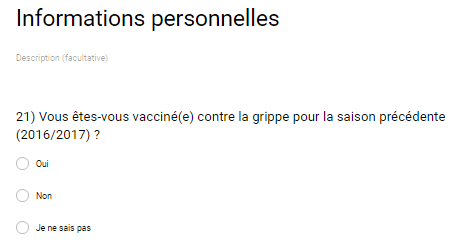


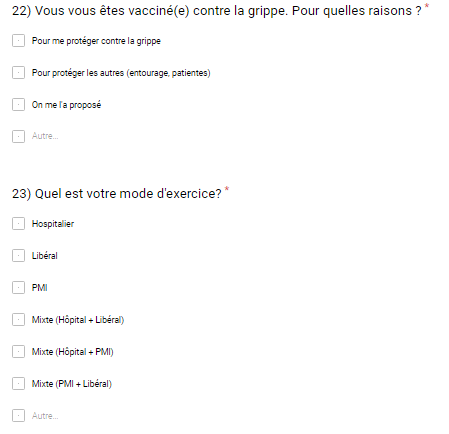


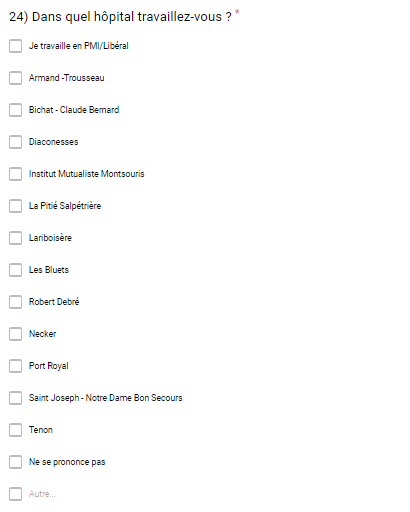


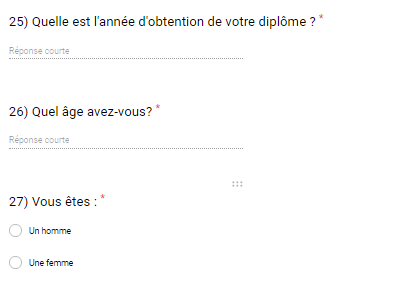

Supplement: S1 File — English and French versions. (DOCX) [file pone.0215251.s001.docx]
